# Exploring the relationship between mental health and urban green space soundscapes: A scoping review

**DOI:** 10.1371/journal.pone.0344125

**Published:** 2026-03-03

**Authors:** Elham Ahmadi, Sophia Baierl, Stephan Voss, Ida Asenkerschbaumer, Ursula Berndt, Leonie Bernhard, Anita Hennig, Anna-Lena Würfele, Michaela Coenen

**Affiliations:** 1 Institute of Medical Information Processing, Biometry, and Epidemiology (IBE), Chair of Public Health and Health Services Research, Faculty of Medicine, LMU Munich, Munich, Germay; 2 Pettenkofer School of Public Health, Munich, Germany; Shenyang Jianzhu University, CHINA

## Abstract

Urban soundscapes, particularly those experienced in green spaces, have been increasingly recognized as factors that influence human mental health. This scoping review explores the existing literature on soundscapes within urban green spaces and their associated mental health outcomes. It aims to classify the methodologies used in this domain, identify mental health outcomes related to urban green space soundscapes, and examine specific soundscape elements and their correlations with mental health. A systematic search of peer-reviewed studies was conducted. After screening titles, abstracts, and full texts, 22 studies met the inclusion criteria. Diverse methodological approaches were identified, with an emphasis on quantitative multi-method designs. Commonly studied mental health outcomes include stress reduction, mood enhancement, perceived restorativeness, and cognitive restoration. Standardized psychometric tools, such as the Perceived Stress Scale (PSS-14), Positive and Negative Affect Schedule (PANAS) and Perceived Restorativeness Soundscape Scale (PRSS) are frequently used as outcome measures. Natural soundscape elements such as birdsong, water sounds, and rustling leaves had a positive association with relaxation and perceived mental restoration throughout all studies, while mechanical sounds, such as traffic noise were linked to adverse mental health outcomes. These findings highlight that natural soundscapes in urban green spaces have a potential positive relationship with mental health by reducing stress and enhancing mood. However, the cross-sectional design and methodological heterogeneity of the included studies limit causal interpretation. Future research should explore multi-sensory experiences and examine soundscapes in diverse urban contexts to provide more robust insights into their relationship with mental health. The practical implications suggest that urban planners should prioritize integrating natural sound elements into urban areas to improve mental health. The study protocol of this scoping review had been registered at OSF (osf.io/4r7gd).

## Introduction

In the past and present, the detrimental health effects of noise have been investigated and discussed thoroughly in various contexts and settings. Although a broad range of scientific literature on noise pollution and its various adverse health effects has been published, the scope of this research is predominantly limited to noise as a one-dimensional variable that focuses solely on negative health outcomes [1]. This narrow approach created a growing interest in investigating sound as a more holistic and subjective concept outside of mere noise-related considerations. The resulting paradigm shift emerged in the form of research which can be described by the umbrella term “soundscape”. First introduced by R. Murray Schaefer in *The Tuning of the World* (1977), the term conceptualizes the acoustic environment as perceived and experienced by individuals [2]. In soundscape research, the acoustic environment is considered as a resource with potential health benefits [3]. As a multidisciplinary field, it is characterized by its focus on the multi-layered acoustic perception of humans. Soundscape research emphasizes the contextual influence of sound sources, auditory perception, and cognitive processes, that impacts an individual’s response and outcomes [4]. Over the last ten years, the International Organization for Standardization (ISO) has developed and published a standardized framework for soundscape assessment using the ISO 12913 series. It collates definitions and guidance regarding data collection and analysis methods [4–6].

A prominent area of current soundscape research is the examination of different soundscape elements and their relationship with health in the context of urbanization. For instance, Aletta et al. systematically explored and identified associations between positive urban soundscapes (e.g., pleasant, calm and less annoying) and health. They reported a faster stress recovery associated with positive urban soundscapes [3]. The current rise in urban soundscape research could be related to a significant increase in the global urban population, from 0.75 billion in 1950 to 4.22 billion in 2018. It is estimated that by the mid-twenty-first century, 68% of the world’s population will reside in urban areas [7]. Urban soundscape research often involves an assessment of different sound sources and their association with health parameters. In recent years researchers have found that natural sounds can reduce stress [8] and improve mood and cognitive performance [9]. While evidence of the relationships between urban soundscapes and health is growing, only recent studies have examined the relationship between mental health and urban soundscapes in the context of urban green spaces. In the literature, exposure to urban green spaces has been associated with a wide array of beneficial health outcomes [10], although the influence of social determinants has not been fully explored [11].

Nonetheless, despite the growing importance of the field, there is no systematic overview of current urban soundscape research trends with an emphasis on green spaces and mental health currently. Therefore, this scoping review aims to map methodologies as well as the investigated associations between soundscape elements (e.g., birdsong, water sounds, traffic noise) and mental health outcomes in urban green spaces. The mapping of evidence could inform public health strategies and urban planning, supporting mental health through the intentional design of restorative green space soundscapes in cities.

## Methods

### Study design

This study follows the ISO 12913 definition of soundscapes as the perceived acoustic environment [4]. Accordingly, sound sources are categorized into sounds of technology (mechanical), nature (natural), or human beings (anthropogenic) [6,12]. No further restrictions were made to capture the full diversity in the literature.

Given that existing reviews only consider the relationship between soundscapes and health in clinical, rural, or urban contexts in a broader sense, a scoping review was identified as the most suitable type of review to provide an overview of the existing scientific landscape. To our knowledge, no review of this type has been conducted or published. The scoping review followed the guidance proposed by the Joanna Briggs Institute [13]. The study protocol had been registered at OSF (osf.io/4r7gd).

The scoping review is based on the methodological framework proposed by Arksey and O’Malley [14], considering later refinement suggestions by Levac et al. [15] and Daudt et al. [16], as well as the recommended enhancements by Westphaln et al. [17]. Furthermore, the preferred reporting items of the PRISMA checklist for scoping reviews were considered [18]. Initially, the framework was constructed using the following five steps: Step 1: Identifying and specifying the research questions, Step 2: Identifying relevant literature, Step 3: Selection of studies, Step 4: Extraction, mapping and charting the data, Step 5: Summarizing, synthesizing and reporting of the results.

### Step 1: Identifying and specifying the research questions

After initially investigating the literature, attention was focused on the following research questions: (1) What methodologies and study designs are used in this domain? (2) What are the different soundscape elements studied? (3) Which mental health outcomes are being studied in connection with urban green space soundscapes? (4) How are the investigated soundscape elements associated with mental health outcomes?

According to the WHO, mental health refers to a state of psychological well-being in which individuals can manage life’s stresses, realize their potential, and function effectively at work and in their communities. It is a crucial aspect of overall health and well-being, embodying more than simply the absence of mental illness [19]. This broad definition of mental health aligns with the definition of mental health used in our scoping review approach, as our rationale is to map the current evidence in a multidisciplinary and integrated approach.

### Step 2: Identifying relevant literature

To investigate the existing literature on the topic of soundscapes and their relationship with mental health, a “Population, Concept, Context” (PCC) framework was employed in order to adhere to the suggestions set out in the JBI Manual and to facilitate clarity in the subsequent stages of the scoping review process [20].

The PCC Framework was adjusted to another context, as the urban population can be described more appropriately with a second context rather than a population itself (Table 1). The search strategy was embedded in the PCC-Framework and was developed and further refined using a team-based approach. The following five databases were applied: *MEDLINE*, *Embase*, *Web of Science*, *PsycINFO* and *LIVIVO.* They were selected to ensure interdisciplinary coverage while avoiding redundancy in line with PRISMA-ScR guidelines. The search strategy was adapted to the respective syntax of the database and was identical for every database search (Table 1). The detailed search strings for all databases are provided in S1 Appendix. There were no restrictions regarding the time of publication. Furthermore, only peer-reviewed articles in English and German were considered for inclusion. Grey literature was excluded in advance, as it is more time consuming to find with systematic search strategies [21]. The search was conducted on the 17^th^ of November 2024. All identified references were uploaded to the citation management software *Endnote 21* (Clarivate; London, UK). Duplicates were removed using *Endnote 21* and *Rayyan* (Cambridge, MA, USA) [22].

**Table 1:** Search strategy.

Category	Search strategy
**Population**	
**Concept**	(sound* OR soundscape* OR natural sound OR auditory environment OR birdsong* OR acoustic environment) AND (mental health OR quality of life OR well* OR restorat* OR stress OR relaxation OR satisfaction OR emotion* OR mood OR perceiv* OR recovery OR psychological OR acoustic comfort OR perception)
**Context 1**	green space* OR park* OR green infrastructure* OR forest* OR garden*
**Context 2**	citizen* OR urban OR city OR cities OR metropolitan

### Step 3: Selection of studies

To develop the eligibility criteria, the PCC Framework was implemented. The model depicts the possible inclusion and exclusion criteria used for the screening. These include eligibility prerequisites for the population, concept and the two contexts (Table 2). Potential differences between studies in real words settings and laboratory studies were suspected; hence, it was decided post-hoc to include only on-site studies.

**Table 2:** Inclusion and exclusion criteria.

Category	Inclusion criteria	Exclusion criteria
**Population**	- Studies on adults (18 + years) - Mixed populations (adults & children) if adult data is analyzed separately - Participants with cognitive impairments, unless exclusive focus is on clinical populations	- Studies only on children/adolescents - Clinical patient-focused studies - Studies solely on tourists
**Concept**	- Focus on soundscape perception and mental health outcomes (e.g., stress, relaxation) - Mental health as secondary focus with plausible connection to soundscapes - Methods like soundwalks or psychoacoustic measures included	- Studies measuring only noise levels (e.g., decibels) - Noise annoyance without subjective soundscape emphasis - No mental health outcome measures - Acoustic environment mentioned but not explored in relation to mental health
**Context 1**	- Conducted in green spaces (parks, forests, gardens) - Mixed green and grey spaces if green space results are separate	- Indoor environments - Built environments without green/natural spaces - Restricted-access green spaces - Grey spaces only
**Context 2**	- Urban/metropolitan areas	- Exclusively rural or wilderness settings - Industrial areas - Lab studies unrelated to urban green spaces - Laboratory experiments simulating urban green spaces (VR or audio-visual stimuli)

Screening was performed using *Rayyan* (Cambridge, MA, USA). An iterative and multi-step team approach was used to conduct the screening. Before the initial title and abstract screening, a test screening of 50 articles was carried out by all team members to revise the eligibility criteria through subsequent discussions within the team. After the test screening, all nine team members executed blinded title and abstract screening against the adjusted inclusion and exclusion criteria. The identified references for abstract screening were divided by nine; hence every team member screened the same proportion of references. The first author (EA) screened all references, as his assessment of inclusion and exclusion functioned as a reference point for discussions of conflicts. A similar approach was applied to the full-text screening. As before, the articles were divided proportionally between team members, with one member reading every article. The last author (MC) performed the final decision on resolving conflicts. To illustrate the selection process, we implemented a PRISMA flow chart with different screening steps.

### Step 4: Extraction, mapping and charting the data

Data extraction was performed by the first author (EA) with a data charting table using *Microsoft Excel* (Redmond, WA, USA). The specific configuration of the extraction table was piloted iteratively through five test extractions, resulting in adjustments in comparison to the first proposed extraction tool in the first version of the study protocol. This corresponds with the recommendations of Pollock et al., who advocated the iterative process of data extraction [23]. The table captures (1) general information, such as authors, publication year, key findings, as well as (2) specific information such as primary mental health outcomes, types of urban green space, and studied soundscape elements.

An openness to emerging subcategories and further classifications was maintained. Thus, we added columns regarding cited theoretical frameworks as well as the seasonal period of the conducted study after the initial test-extraction.

### Step 5: Summarizing, synthesizing and reporting of the results

Results were synthesized and organized in alignment with the research question and presented across three comprehensive extraction tables. The first table provides an overview of the preliminary study details, whereas the second describes more specific methodologies and population characteristics. The third table was dedicated to key findings, limitations and practical recommendations. The tabular data was further complemented by a narrative summary to provide context and highlight key observations made during extraction. Furthermore, the results were structured into four distinct parts to account for the specific aims (research questions; RQ) outlined in the scoping review: RQ (1) Study design and general methodology; RQ (2) Acoustic dimension and studied sound sources; RQ (3) Conceptualization and measurement of mental health outcomes and RQ (4) Soundscape elements and their associations with mental health. Figures were generated using *Python* [24]. Maps were created using *Basemap 2.0.0* [25], which incorporates data from the *Generic Mapping Tools (GMT)* [26] and the *Global Self-consistent, Hierarchical, High-resolution Geography Database (GSHHG)* [27]. Both datasets are distributed under the GNU Lesser General Public License (LGPLv3) [28].

## Results

**Fig 1:**
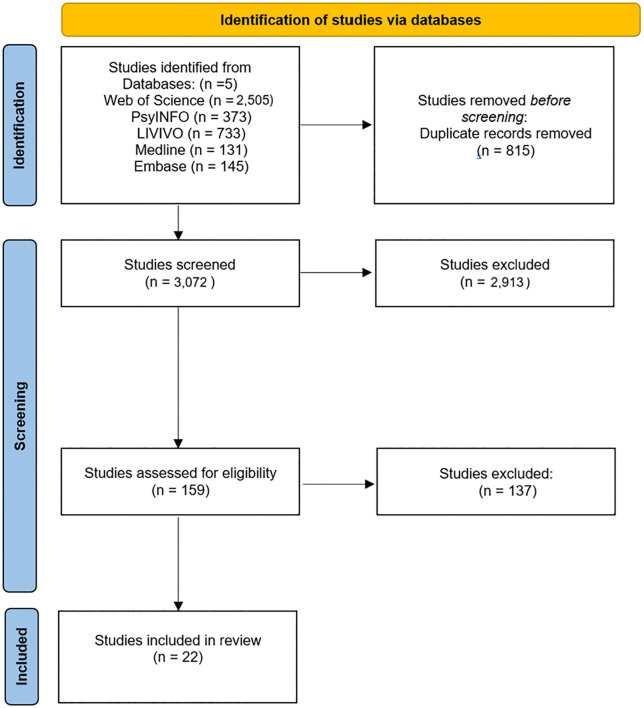
PRISMA flow chart for scoping reviews PRISMA 2020 statement.

A total of 3,892 articles were identified through the database searches. After removing duplicates with Endnote and Rayyan*,* 3,072 articles remained for title and abstract screening. Of these, a total of 159 articles were identified for the full-text screening. After full-text screening and resolving all conflicts, 22 articles remained (Fig 1).

### Overview of included studies

**Fig 2:**
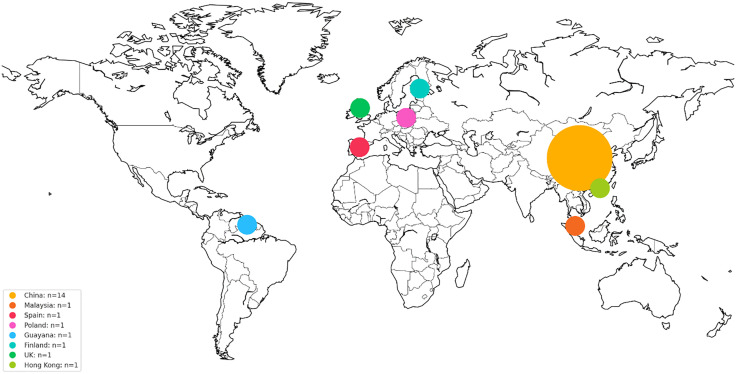
World heatmap showing the location of the conducted studies (n = 22). Created in *Python* using *Basemap 2.0.0* [25] including coastline and boundary data from the *GMT* [26] and *GSHHG* [27] under LGPLv3 [28].

**Fig 3:**
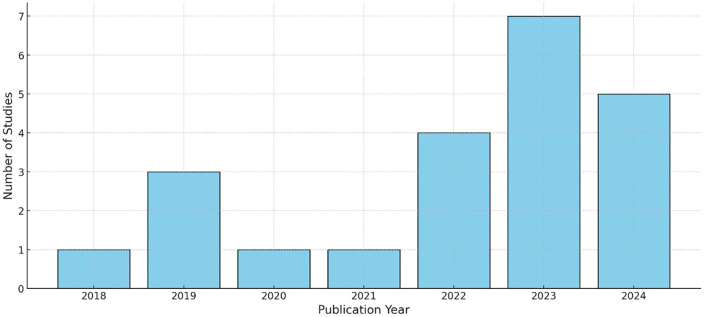
Publication year of included studies (n = 22).

With 14 of the 22 included studies, China accounted for nearly 70% of the conducted studies overall (Fig 2). Furthermore, the earliest study was conducted in 2018, whereas 17 studies were published after 2020 (Fig 3). The research settings predominantly involved urban parks (n = 13) and urban forests (n = 6). Further details of the included studies are shown in Table 3.

**Table 3:** Overview on study methods and population (n = 22).

Study Method	n (%)
Quantitative	19 (82)
Qualitative	0 (0)
Mixed	3 (18)
**Population**	
Average Sample Size	467
Age considered	
Yes	8 (36)
No	14 (64)
**Gender considered**	
Yes	18 (82)
No	4 (18)
**Education level considered**	
Yes	9 (41)
No	13 (59)
**Employment considered**	
Yes	2 (9)
No	20 (91)

**Table 4:** General information about the included studies (n = 22).

First Author (Z-A)	Year	Country	Objective of the study	Methodology	Description of green space
Zhu, Y. [31]	2023	China	To analyze the relationship between source perception, soundscape perception, restorative perception, and health benefits, and to model the relationships between soundscape perception, restorative perception, and health benefits using structural equation modeling (SEM)	Field experiment conducting soundwalks, questionnaire surveys, and Structural Equation Modelling (SEM)	The park offers diverse landscapes, including ancient trees, temples, and healing trails, attracting visitors for forest bathing experiences. The three km healing trail consists of ten soundscape healing points across various gardens, including botanical gardens, waterside spaces, and open lawns, each providing distinct natural soundscapes to promote relaxation and well-being. Altogether, the park covers approximately 390 ha.
Zhou, Y. [32]	2022	China	To explore how different types of soundscapes in urban parks affect citizens’ emotions using sound pressure level measurements and questionnaires	Soundscape experiments using sound pressure level meters, questionnaires and structural equation modeling (SEM). Seasonal differences between summer and winter were analyzed	Beijing Sun Park (288.7 ha, Chaoyang): A large comprehensive park that supports diverse outdoor activities. It offers recreational areas for cultural entertainment and children’s activities, resulting in a soundscape dominated by living sounds like people talking and playing; Olympic Forest Park (680.0 ha, Chaoyang): The largest ecological park in Beijing, preserving a natural state with minimal human interference. It features abundant natural sounds such as bird calls and rustling leaves, providing a tranquil environment for visitors to connect with nature and relax; Beihai Park (71.0 ha, Xicheng): A cultural heritage park with strong educational and tourism functions. It is popular for family visits and leisure activities, with a balanced soundscape of natural, living, and cultural sounds; Ditan Park (37.4 ha, Dongcheng): Another small cultural heritage park, known for its parent-child educational activities and quiet rest areas. Average soundscape with no specific sound type dominating.
Zhao, W. [30]	2020	China	To evaluate the effects of different types of bird song using the Perceived Restorativeness Soundscape Scale (PRSS) during different seasons and at different heights in an urban park	On-site questionnaire and sound playback experiments across seasons and heights	Sun Island Park covers 3,800 ha and features diverse landscapes, including deciduous and coniferous forests, shrubs, and grasslands, with waters that provide habitats for insects and birds. The park’s eight selected sites, such as Rabbit Island, Swan Lake, and Sun Waterfall, offer rich biodiversity, promoting bird-related soundscapes and enhancing the interaction between visual and acoustic restorative experiences. The sites are well-connected and easily accessible, supporting high ecological and recreational value.
Zhang, T. [33]	2019	China	To examine the relationships between multi-sensory perception and mental restoration in urban parks	Mixed-methods survey (on-site and online), structural equation modeling	70.7 ha urban park with trees, shrubs, lawns, lakes, hills; green coverage of 90% including zones for flowers, entertainment, elderly activities, and rest
Zhang, J. [34]	2023	China	To examine the effects of different acoustic environment types in urban parks on stress relief using ECG analysis	Field study with physiological (ECG) and psychological (PSS-14, PANAS) stress indicators; short-term transient heart rate and Root Mean Square of Successive Differences analysis	Green Space with trees, water features, surrounded by residential and mixed-use properties, divided into activity zones
Yin, Y. [35]	2023	China	To explore human behavior and emotional response to visual and aural contact with urban nature	Multi-methods approach with soundwalk, QGIS and space syntax on-site mapping and questionnaires	Encompasses 132 ha and showcases a variety of natural settings, including lakes, forests, herbs, and plants. The garden is a favored place for recreation, sightseeing, and family engagements, providing an array of anthropogenic and natural noises; Grasslands constitute 51.9%, followed by forests at 23.7% and waterscape at 6.7%, while hardscape elements, including roads, squares, and playgrounds, account for around 17.7% of the overall area.
Wu, Y. [36]	2023	China	To examine how various physical, psychological, and environmental factors influence visitor emotions in urban green spaces	Questionnaire-based survey, real-time environmental monitoring, path analysis	Parks included diverse landscape styles (e.g., lakes, woodlands, modern and classical gardens) and varied in size from 3.43 to 42.51 ha. The study captured real-time data on thermal, air, and sound environments across different park settings.
Tian, L. [37]	2023	China	To propose strategies for optimizing soundscape quality by correlating physical parameters with human perception	Soundwalks, Sound Pressure Level measurements, GIS spatial analysis	21.6 ha park with 69.4% water bodies and various features such as lakes, vegetation, walking paths, and recreational spaces
Payne, S. [29]	2019	UK	To investigate the psychological restorative effects of visiting urban quiet areas and their association with perceived soundscapes	Cross-sectional survey using a soundwalk approach. Expert Assessment with European Quadmap’s tool	The green spaces include small and medium-sized urban parks/gardens with features such as lawns, trees, shrubs, gravel paths, water bodies, and pathways for recreation; Dunbar’s Close Garden: 0.19 ha, Palmeira Lawns: 0.72 ha
Marafa, L. [38]	2018	Hong Kong	To evaluate perceived tranquility in seven green spaces of Hong Kong and understand factors that enhance or diminish tranquility in urban and country park settings	Mixed-methods approach: quantitative data collection via questionnaires assessing tranquility, anxiety levels, and usage patterns, coupled with qualitative data (open-ended questions)	Urban parks with moderate greenery and recreational amenities, Kowloon Park (13.5 ha) with rugged terrain providing recreational and cultural amenities, Shatin Central Park (8.1 ha) with gardens and playparks
Li, W. [39]	2024	China	To explore whether differences exist in the perceived soundscape affective perception quality and visual landscape quality under audio-visual interaction in different types of parks, and whether these differences affect perceived restorativeness	Multi-method approach: Field surveys conducted in urban parks combined with soundscape recordings	North city sports park: 45 ha; Canal Asian Games Sports Park: 46.7 ha; Yile Park: 5.1 ha; Riverside Park: 4.3 ha; Hangzhou Forest Park: 5.5 ha; Xiaoqiao Creek ecological Park: 5.4 ha; Xixi National Wetland Park: 1,150 ha; The parks offer different vegetations, layouts and recreational purposes.
Li, S. [40]	2023	China	To explore the effects of urban forest environments on psychological restoration	Natural experiment conducted in three urban forest parks, with assessments using questionnaires and physiological indicators	Fenghuangling Forest Park: rich in natural landscapes, planted with pines, cypresses, ginkgoes and mountan apricots Yangtai Mountain Forest: cypresses, ginkgoes, and maples Baiwang Mountain Forest Park: greasy pines and cypresses
Lee, J. [41]	2023	Malaysia	To investigate how a park’s soundscape affects subjective well-being of park users based on objective measurements of sound level, and soundscape descriptors	On-site assessments in the selected parks for obtaining information on the acoustic readings and subjective responses to the acoustic environment, sound level measurements	KLCC Park: 20.2 ha; Taman Tasik Permaisuri: 48.6 ha; Bukit Jalil Recreational Park: 32.4 ha; Putrajaya Botanical Garden: 93.1 ha; Parks with diverse features, including sports and recreational facilities, different landscape, man-made ponds, and a golf resort and botanical gardens
Lan, Y. [42]	2024	China	To address the research gap on the impact of outdoor visual–acoustic interactions on public response	Field surveys and quantitative analysis	Jinniushan Sports Park: “Characterized by undulating terrain and picturesque scenery, it is the largest community-type semi-hilly sports park in the heart of Fuzhou City“; Fuzhou National Forest Park (also known as “Fuzhou Botanical Garden”) is the first national-level forest park in Fujian Province, one of the top ten forest parks in China, and one of the six 4A-level scenic spots in the Fuzhou area. The total area spans 2,891.3 km.
Korpilo, S. [43]	2024	Finland	To evaluate the difference between physiological and psychological restoration potential of urban green and blue spaces	Field-based quasi-experiment with multisensory data collection (questionnaires, physiological measurements, soundscape evaluations)	Kalastama urban park: surrounded by residential buildings, shopping and cultural centers, with grass, shrubs and young trees as vegetation; Mustikamaa forest site: pine-dominated remnant forest and rocks, high biodiversity.
Jin, T. [44]	2024	China	To investigate the integrated effects of visual and aural stimuli from urban nature on human behavior and emotional responses, especially in the context of traffic noise influence	Field-based observational study using questionnaires, soundwalks, GIS mapping, audio-visual landscape surveys, and sound pressure level recordings	One-third of the park is covered by water, showcasing a variety of landscape components, such as aquatic areas, lawns, forests, and paved surfaces. It primarily fulfills recreational, sightseeing, and ecological conservation purposes.
Jaszczak, A. [45]	2021	Poland	To assess the influence of soundscapes on well-being and develop park redesign criteria derived from study’s findings	Mixed-methods study combining sound pressure level measurements, user interviews, expert interviews and preparation of schemes for re-design of the park	8 ha park with old deciduous trees, exotic plants, lawns, shrubs, and recreational areas (e.g., lake, sports center)
Herranz-Pascual, K. [46]	2019	Spain	To determine the influence in urban open public spaces of the sound environment and its perception (soundscape) on users’ health	Observational study using a smartphone-based toolkit to assess soundscape and emotional states	Salinillas Urban Park: 90% green spaces; Olarizu Urban Park: 80% green spaces
Guo, Y. [47]	2022	China	To investigate if the forest park soundscape has an impact on relieving mental stress	Questionnaire-based field study and statistical analysis	Subtropical monsoon climate; diverse landscapes with functional areas such as bamboo forest, reservoir, streams, stone steps, and cultural pavilions, divided into waterfront landscape, plant landscape and cultural landscape area; 410 ha
Guo, X. [48]	2022	China	To investigate how the various dimensions of soundscape (perception, pleasantness and eventfulness), affect visitors’ perceived soundscape restorativeness in the selected urban parks	Questionnaire-based field study consisting of three parts (Sociodemographic information, Russels Circumplex Model Questionnaire, Perceived Restorativeness Scale Questionnaire)	West Lake Park (42.51 ha) is a classic Chinese garden with almost three-quarters covered by water features; Zuohai Park (35.47 ha) is a “five continents scenery” theme park with an amusement section and an activity center for senior citizens; Wenquan Park (10 ha) follows a “European style” with rich dynamic water features. Jinan Park (68.27 ha) presents a modern urban design, including several ecological engineering measures; Fuzhou National Forest Park (859.33 ha) provides rich plant resources and multiple recreation and science education opportunities.
Guo, X. [49]	2024	China	To analyze how soundscape elements in urban parks influence perceived restorative experiences and visitor behavior	Questionnaire-based field study consisting of four parts (Sociodemographic information, Russels Circumplex Model Questionnaire, Perceived Restorativeness Scale Questionnaire, questionnaire regarding visitors’ behavior)	West Lake Park (42.51 ha) is a classic Chinese garden with almost three-quarters covered by water features. Zuohai Park (35.47 ha) is a “five continents scenery” theme park with an amusement section and an activity center for senior citizens; Wenquan Park (10 ha) follows a “European style” with rich dynamic water features; Jin’an Park (68.27 ha) presents a modern urban design, including several ecological engineering measures; Fuzhou National Forest Park (859.33 ha) provides rich plant resources and multiple recreation and science education opportunities.
Fisher, J. [50]	2022	Guayana	To explore how perceptions of different sounds, like perceptions of bird species richness, perceived naturalness and concerns for personal safety all contribute to the perceived restorativeness of the green/blue spaces, and whether perceived restorativeness acts as a mediator of people’s wellbeing	Multi-methods approach combining surveys, acoustic recordings, and biodiversity assessments. The study used sound meters and bird point counts to gather environmental data, and self-reported questionnaires to measure perceptions and wellbeing outcomes	The green spaces varied in size and features, with some areas featuring dense vegetation and water bodies, while others were more urbanized and artificial.

The investigated green spaces showed vast differences in size. For instance, “Dunbar’s Closed Garden” in Edinburgh, Scotland only encompasses 0.19 ha [29], whereas Sun Island Park in Harbin, China spans 3,800 ha in size [30] (Table 4).

The sample sizes displayed considerable differences, with small exploratory cohorts and larger population-based analyses (Table 4). The smallest sample yielded 20 participants [37], whereas the largest sample included 1,161 participants [39]. Most studies were conducted during the summer months (n = 15). Only two studies included both warm and cold seasons.

### RQ (1): Study design and general methodology

The identified studies demonstrate the use of diverse techniques to capture objective sound metrics, subjective soundscape perception and psychometric parameters. All studies were based on a cross-sectional design, in which participant characteristics were analyzed at a single point in time. Nonetheless, data collection was conducted throughout multiple days and times of day, with an emphasis on mornings and afternoons [32,41,46]. Fifteen studies were conducted during summer/spring, while three were conducted during autumn/winter. Eleven studies examined more than one green space. Nineteen of the 22 studies implemented a quantitative multi-method approach, where the measurement of Sound Pressure Level (SPL) was combined with questionnaires regarding the subjective assessment of the acoustic environment as well as questions related to acoustic perception, mental health and well-being. Only three studies used a mixed-methods design. For instance, Jaszczak et al. employed qualitative interviews with participants and experts using a mixed-methods approach, to capture the nuances of individual soundscape experiences [45]. Payne and Bruce implemented qualitative content analysis to generate themes from their questionnaire with open-ended questions [29]. Furthermore, 21 studies collected data on sociodemographic characteristics. Table 4 further summarizes study methods and sociodemographic characteristics.

Statistical methods across the reviewed studies varied considerably, ranging from basic descriptive analyses (n = 22) to more advanced approaches, such as regression analyses (n = 8), ANOVA (n = 5) and structural equation modelling (SEM) (n = 7). For example, Y. L. Guo et al. combined Spearman’s rho, Kruskal–Wallis tests, and hierarchical multiple regression to link social and demographic factors with stress-related outcomes in park visitors [47]. Apart from that, X. Guo et al. used SEM with Confirmatory Factor Analysis (CFA) to examine how perceived pleasantness and eventfulness influence restorativeness [48]. Herranz-Pascual et al. and Payne and Bruce relied on ANOVA to compare mental health parameters across different urban environments [29,46].

### RQ (2): Acoustic dimension and studied sound sources

Most of the authors of the included studies (n = 13) had a similar approach. They categorized sound sources into three overarching sound domains: natural (e.g., birdsong, water sounds), mechanical (e.g., traffic, construction noise), and anthropogenic or social (e.g., conversations, laughter), with slight adjustments in the scope of applied categories.

Four studies measured Sound Pressure Levels (SPL). Tian et al. additionally paired SPL with Geographical Information Systems (GIS) mapping to visualize soundscapes on a map [37]. Seven studies were based on soundwalks, while two studies implemented the Normalized Difference Soundscape Index (NDSI). The NDSI was used to measure the balance between acoustic biodiversity and anthropogenic disturbance [51]. Furthermore, the soundwalk method was applied as an empirical approach to identify soundscapes and their components. The fundamental objective of a soundwalk is to motivate participants to engage in attentive listening and formulate assessments of the auditory stimuli encountered [52]. It was also one of the more frequently applied method within the included studies (n = 7) to collect data for exploring areas of human response to an acoustic environment. The soundwalk method was standardized according to the International Organization for Standardization (ISO-12913–2). While plenty of the included studies implemented the ISO-12913–2 Guidelines (n = 6), most of the studies adjusted the methodological application specified in ISO/TS 12913−2. For example, Zhu et al. expanded sound source identification using a 5-point Likert scale with two additional points [31].

### RQ (3): Conceptualization and measurement of mental health outcomes

A recurring theme in all studies is the recourse to similar theoretical frameworks. One of these theories is the highly influential Attention Restoration Theory (ART) introduced by Kaplan and Kaplan (n = 20). It states that mental focus can be restored by reducing fatigue through exposure to the natural environment. Restorative environments must provide four components: fascination (elements that effortlessly capture attention without cognitive strain), extent (an environment that feels immersive and expansive), compatibility (a match between the environment and an individual’s needs or preferences) and a sense of being away (a sense of psychological distance from routine or stressful environments) [53]. Another theoretical framework prevalent throughout a multitude of studies (n = 13) is the Stress Reduction Theory (SRT) by Ulrich. It proposes that humans have an inherent evolutionary-based preference for natural settings, which are perceived as safe and supportive for survival. According to the SRT, viewing or experiencing nature triggers a positive emotional response, leading to reduced stress levels and improved mood [54]. Furthermore, thirteen studies used both theories as their theoretical framework. Only one study referenced no theoretical framework.

To evaluate participants’ perceptions of the restorative potential of urban green spaces, Yue Ma et al. and Wu et al. implemented the Perceived Restorativeness Scale (PRS), with its items based on ART [36,39,55]. Another prominent tool for measuring restoration in natural environments was the Perceived Restorativeness Soundscape Scale (PRSS) developed by Payne (n = 4). Although it is also based on ART, it is more focused on adapting the concept to auditory environments [56].

Other standardized questionnaires and scales to measure stress reduction and emotional responses were also included. Zhang et al. used the Perceived Stress Scale (PSS-14) to screen participants for psychological stress before participating in the study, while also implementing the Positive and Negative Affect Schedule (PANAS) to measure short-term mood changes and emotional recovery during changes in the acoustic environment [34,57,58]. Furthermore, some studies (n = 3) introduced physiological methods to assess stress markers, such as heartrate monitoring and skin conductivity measurements [34,42,43].

### RQ (4): Soundscape elements and their associations with mental health

Overall, the studies showed a high degree of heterogeneity in their investigated relationships between soundscape elements and mental health. A recurring pattern throughout the included studies was the consistency in reporting positive relationships of natural soundscapes with different mental health parameters, despite different strategies in study designs and investigated soundscape elements/mental health parameters (n = 13). This was the case for both direct and indirect variables associated with mental health. Tian et al. reported improved relaxation and pleasure perception [37], whereas Zhang et al. reported reduced heart rate and heart rate variability indicating mental stress relief through natural sounds [34]. Lee et al. further contributed to this by covering similar results for the relationship between urban park natural soundscapes and well-being in Kuala Lumpur and Putrajaya [41].

The most frequently examined natural sounds included birdsong, water sounds and wind sounds. There was an emphasis on birdsong, as numerous (n = 9) of the included studies associated birdsong as a crucial part of urban green space soundscapes. The positive relationship between mental health and birdsong was reported in a variety of studies, including associations with relaxation, mental stress relief and PRS [31,34,39,47,50]. Zhao et al. supplemented these findings with investigations regarding the association of different bird species with the PRS and the inclusion of seasonal variance. They found that woodpecker and sparrow sounds had high PRS scores throughout summer and winter, whereas crow sounds were rated the lowest during both seasons [30]. Furthermore, higher perceived bird species richness had a positive relationship with the PRS and higher well-being [50]. Correspondingly, positive mental health associations with water and wind sounds were also reported consistently within a large proportion of studies [31,32,34,39,44,47].

Anthropogenic soundscapes had mixed results, with some studies indicating positive and others reporting negative mental health associations. Tian et al. stated that the perceived occurrence of human activity sounds has a significant negative correlation with pleasure and relaxation [37]. In contrast, Zhou et al. indicated that living sounds such as activities, laughter, and children frolicking have a positive association with emotions [32]. Li and Liu reported that social sounds had a positive relationship with the PRS through sounds like playing children, while loud conversations had a negative relationship [39].

**Fig 4:**
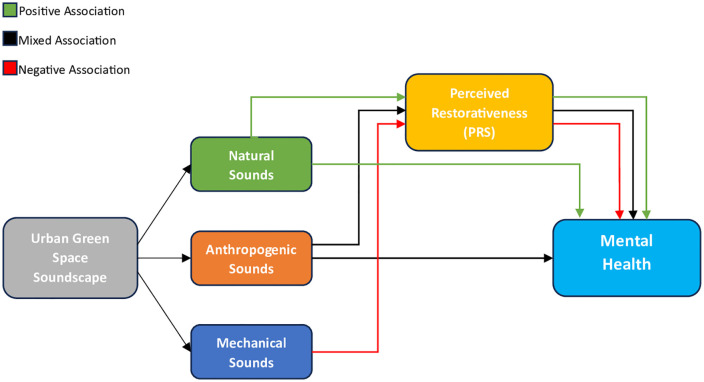
Simplified model of associations between soundscape elements and mental health based on synthesized results.

The results for the associations of mechanical sounds with mental health were uniform throughout the included literature. Negative relationships have been reported across multiple studies, through various direct and indirect mechanisms. Mechanical sounds were associated with impeded recovery from mental stress [34], reduced perceived restoration [39] and lower well-being [32]. This was especially the case for traffic sounds, which were rated negatively throughout the studies [34,35,41]. Fig 4 illustrates the reported relationships across the included studies.

For further information on population characteristics and specific methodologies as well as key findings of the included studies see S1 and S2 Tables.

## Discussion

With this scoping review, the existing body of literature on the relationship between mental health and soundscapes embedded in an urban greenspace context was mapped. The different methods were illuminated and approaches the authors applied and gained a more comprehensive understanding of the different investigated soundscape elements and their associations on mental health.

### Study design and general methodology

Although the initial exclusion of laboratory studies influenced the heterogeneity of the reviewed studies, there were still variations in the applied study design approaches. It was shown that the reviewed literature uses different methodologies, with an emphasis on cross-sectional [29,43,44] and multi-methods designs [38,39,50]. Only a small fraction of the studies took temporal changes into account; Zhao et al. and Zhou et al. postulated better mental health outcomes in the summer months compared to winter [30,32]. The lack of seasonal variance analysis could carry a risk of overestimating the restorative potential of urban green spaces, as summer months include conditions with a possible positive influence, such as favorable weather and higher levels of natural sounds [59].

Although the reviewed studies showed great varieties in statistical methods and sample sizes, some studies resorted to convenience samples [37], while other studies specifically aimed at recruiting university students [42] despite college students not necessarily being able to represent the general public [20]. Moreover, only three studies used a mixed-methods design. The lack of qualitative methods seems to be a missed opportunity, as they can reveal important contextual meanings, emotions, and personal experiences, giving urban citizens an opportunity for involvement in the research process [60]. This approach would also align more with the original soundscape paradigm description of Schaefer, which emphasizes the “individual*”* acoustic experience [2].

Most of the included studies were published in China and after 2020. This surge in health-related studies embedded in environmental contexts could be part of the “Healthy China 2030” national strategy, which aims to improve the overall health of the population by setting targets for sustainable development until 2030 [61]. This geographical bias poses a challenge for the generalization of the results of this review. Prior research has found that the socio-cultural context influences individual’s perception of auditory environments [62–65]. Cross-national studies have shown that participants from different nations vary in their preferences for human or natural sounds [66,67], in how they link pleasantness and vibrancy to these sound categories [66,68], and in how they associate sound levels with liveliness or noise [69,70]. Due to their multidimensionality and context-dependence the socio-cultural influences are difficult to quantify [63]. Along with the scarcity of research from other regions, particularly low-income countries, this poses a challenge for cross-cultural comparison and affects the generalizability of the associations found between soundscapes elements and mental health in this review.

It is not necessarily clear what qualifies as an “urban green space”, as there might be some fundamental differences in the definition of terms. While Chinese studies tend to refer to outer skirt, large-scale forest/park environments as “urban parks” [30,39], studies from Europe have applied the term to more small scaled green spaces within the city [29,46]. Additionally, most of the included studies investigated more than one green space, complicating comparability across studies [29,32,38–42,46,48–50]. Global geographical differences in the flora and fauna should also be considered, as they were not addressed in the included studies.

### Acoustic dimension and studied sound sources

While most of the included studies categorized soundscape elements into similar categories (natural, anthropogenic and mechanical), it is not entirely clear how the specific categorization into different sound sources is justified. Such heterogeneity in classification has also been noted in broader soundscape literature. For example, Brown et al. and Kang et al., highlight how inconsistent category boundaries and labeling complicate direct comparisons across studies, limiting opportunities for meta-analyses and the development of shared theoretical frameworks [71,72]. While ISO 12913−1 through ISO 12913−3 offer guidelines for terminology and categorization, researchers often apply these methods to specific study contexts or participant populations, thereby creating subtle (and sometimes major) deviations in practice. The strict compliance with ISO/TS 12913−2 guidelines is therefore rather limited, which is also an observation Aletta and Torresin made within their literature overview regarding ISO/TS 12913−2 data collection compliance [73].

Moreover, the social and cultural contexts of a given location can further influence classification. What one group perceives as “pleasant anthropogenic” (e.g., children playing) may be deemed intrusive or mechanical by another, complicating attempts to develop a universal taxonomy. Jaszczak et al., who combined interviews with experts and park visitors, demonstrated how qualitative approaches can help uncover nuanced perceptions of sound [45]. Similarly, Payne and Bruce used qualitative content analysis to generate open-ended themes, suggesting that top-down categorizations (e.g., natural vs. anthropogenic) do not always capture the lived or contextual experiences of sound [29].

### Conceptualization and measurement of mental health outcomes

The prominence of ART [53] and SRT [54] in the included studies highlights the consistent relevance of these foundational frameworks in explaining how urban green space soundscapes promote mental health. In line with Kaplan and Kaplan’s concept of cognitive restoration, the PRS [36,39,55] and PRSS [56] were used across multiple studies to quantify how specific auditory features, such as birdsong, wind, or running water, can provide fascination, a sense of being away, and other restorative components. These attributes align with broader systematic reviews suggesting that exposure to natural environments can have a positive impact on mental health outcomes [74,75].

Parallel to ART, the SRT has provided a foundation for understanding the affective and physiological pathways through which natural exposure reduces stress. Research within this review revealed increasing use of objective stress markers, including heart rate variability and skin conductance [34,42,43], suggesting a shift towards biopsychosocial approaches that capture complex mental health outcomes outside of psychometric instruments. Hume and Ahtamad also suggest that using physiological markers alongside subjective evaluations of individual sounds offers a more comprehensive, objective means of assessing soundscapes [76].

Nevertheless, both our review and the general body of literature reveal methodological heterogeneity: researchers deploy a range of measures (PRS, PSS, PANAS and PRSS), as well as various physiological measurements. This diversity is associated with the multidisciplinary nature of soundscape research (e.g., environmental psychology vs. public health vs. urban planning) and can complicate cross-study comparisons. Consequently, this echoes critiques in environmental psychology that call for standardized outcome measures or at least clearly documented assessment protocols [72].

### Soundscape elements and their associations with mental health

The findings from this review underline the restorative potential of natural soundscape elements which are consistently associated with reduced stress, enhanced mood and greater perceived restorativeness [31,33,37]. This was especially the case for birdsong, although there may be differences in the restoration potential between bird species [30]. These differences were also observed by Ratcliffe et al., who found that not all birdsongs were regarded as restorative [77]. Similar results were reported for water- and wind- related sounds. Lan et al. additionally highlighted the “sound-masking” properties of urban forest waterfronts [42]. These results further contribute to a growing body of sound-masking literature, which focuses on noise mitigation through water sounds [78–81].

Overall, these reported outcomes align with broader evidence showing that natural sounds can facilitate stress recovery [82,83]. However, the exact mechanisms behind these benefits may differ across cultural contexts, as individual preferences and sociodemographic backgrounds may mediate these associations. To account for this potential bias, a substantial portion of the studies collected sociodemographic information [32,46,47]. While information on age and gender was omnipresent, only a few of the included studies collected information on income and education. Lee et al. found that age and education, followed by park distance, sensitivity of park users as well as gender and occupation might influence soundscape perception. Thus, sociodemographic information should be collected more rigorously [84]. We assume that it can be challenging to determine whether a person’s response stems directly from the environment or is partly shaped by existing vulnerabilities or underlying health conditions. Moreover, the observed associations may not result solely from the sound itself; instead, it could be influenced by intervening factors such as overall health status or sleep disturbance [85].

Anthropogenic and mechanical soundscape elements yield mixed or negative results, with loud traffic consistently undermining psychological restoration [39,81]. These findings align with concerns raised by the WHO regarding chronic noise exposure and mental well-being [86]. Further classification of the relationship between traffic noise and mental health seems challenging, as the associated body of literature does not provide clarity. For instance, Hegewald et al. found no significant relationship between road noise and anxiety/depression [87], whereas Dzhambov and Lercher pointed out a potential link [88]. Notably, the current trend in soundscape research is to consider soundscape elements and their associated health parameters individually. Bai and Zhang expanded this narrow approach by introducing multi-element natural soundscapes, indicating that combined natural soundscapes have a higher restorative effect than single-element natural soundscapes [89]. Therefore, future research should advance the multi-element soundscape approach to gain a more holistic understanding of urban green space soundscapes.

Although our review focused primarily on auditory aspects, several studies indicated that integrating multisensory elements, combining visual with auditory inputs, can improve our understanding of soundscape experiences [35,42,44,48]. The corresponding body of literature suggests that early crossmodal interactions, particularly when emotional cues are present, may heighten attention and arousal, offering more differentiated insights than auditory data alone [90,91]. Shao et al. identified multiple correlations between visual attributes, aural attributes and sound sources, influencing perceived soundscape comfort [92]. According to experimental studies, soundscapes are rated more positively when accompanied by visual stimuli and audiovisual stimuli are more beneficial to mental health than auditory or visual stimuli alone, particularly when the visual and auditory components are congruent [93,94]. These findings highlight the need for research on multisensory or multimodal environmental perception to comprehensively understand the pathways through which urban green spaces influence mental health.

### Strengths and limitations

One of the strengths of this scoping review is the team-based approach during the screening phase of the literature as well as the inclusion of multiple databases due to the multidisciplinary character of soundscape research. Another strength is the broad research strategy, as studies from different disciplines were included, thus allowing for a multidisciplinary scope. The compliance with Arksey and O’Malley’s framework as well as the use of the PRISMA-ScR-Checklist is considered as a strength as well.

However, this study had some limitations. Both the pilot testing of the data extraction and the original data extraction were conducted by only one person, thus deviating from the JBI recommendations regarding data extraction [23]. Although a broad search strategy was employed, limiting the language to English and German might have introduced bias. We also did not include grey literature, which could have provided additional perspectives for this review. The lack of stakeholder inclusion may also have reduced the depth of contextual insights, as local expertise in acoustic environments can signify specific knowledge about certain areas [95]. Conclusively, heterogeneity in methodologies posed a challenge for drawing firm conclusions across studies.

## Conclusion

This scoping review provides the first comprehensive overview of current research on the associations between urban green space soundscapes and mental health. Most studies assessed soundscapes through both SPL and subjective evaluations, yet only a subset adhered to the methods and sound specifications of the ISO 12913 standard. A variety of mental health outcomes, based on frameworks such as ART or SRT, was examined, including stress reduction, perceived restoration, and affect, using instruments such as PRS, PRSS, PANAS, and physiological stress markers. The model presented in this review provides an overview of the observed relationships between urban green space soundscapes and mental health outcomes, contributing to a more structured understanding of the current evidence in this emerging research area.

We found that natural soundscape elements, particularly birdsong, were consistently associated with improved mental health outcomes (reduced stress, enhanced restoration and better overall mental well-being). The results for anthropogenic sounds were mixed, whereas mechanical sounds were rated negatively. However, the cross-sectional design and methodological heterogeneity of the studies limit comparability and preclude causal inference. The predominance of studies conducted in China further highlights a geographic imbalance and limits generalizability. The scarcity of research from low-income countries reveals an evidence gap, indicating the need for further studies in these regions to draw broader conclusions about the relationships between soundscapes and mental health.

The findings underscore the need for applying standardized soundscape classification and assessment procedures to improve comparability across studies. Contextual factors such as seasonal variation, cultural influences, and spatial characteristics could play critical roles in shaping how urban soundscapes affect mental health, emphasizing the importance of longitudinal and multisensory research approaches that capture temporal and contextual variation.

Understanding the links between acoustic environments in urban green spaces and mental health is increasingly relevant as urbanization progresses. By mapping existing evidence and highlighting methodological gaps, this review provides a foundation for more comprehensive, interdisciplinary research on this topic. The insights inform public health strategies and urban planning policies aimed at designing restorative urban green spaces that support mental health.

## Supporting information

S1 Appendix Detailed search strategy for all databases. (DOCX)

S1 Table Population characteristics and specific methodologies of included studies (n = 22). (PDF)

S2 Table Key findings, limitations and practical recommendations of included studies (n = 22). (PDF)

S1 File Study protocol. (PDF)

S2 File Preferred Reporting Items for Systematic reviews and Meta-Analyses extension for Scoping Reviews (PRISMA-ScR) Checklist. (PDF)
