# Supplementary material for: Exploring the relationship between mental health and urban green space soundscapes: A scoping review
Source: PLoS One. 2026 Mar 3;21(3):e0344125. doi: 10.1371/journal.pone.0344125 (PMC12956135; doi:10.1371/journal.pone.0344125)
Supplement: S1 Appendix — (DOCX) [file pone.0344125.s001.docx]

**S1 Appendix. Detailed search strategy for all databases.**

The searches were conducted on **November 17, 2024** in all databases.
Detailed search strings for each database are provided below.

**Web of Science (n=2.505 results)**

All=((sound* OR "auditory" OR birdsong* OR "acoustic")

AND (mental OR "quality of life" OR well* OR restorat* OR stress OR relaxation OR satisfaction OR emotion* OR mood OR perceiv* OR recovery OR psychological OR comfort OR perception)

AND (green space* OR park* OR green* OR forest* OR garden*) AND (citizen* OR urban OR city OR cities OR metropolitan))

**PsycINFO (n=373 results)**

TX (sound* OR auditory OR birdsong* OR acoustic)

AND TX (mental OR "quality of life" OR well* OR restorat* OR stress OR relaxation OR satisfaction OR emotion* OR mood OR perceiv* OR recovery OR psychological OR comfort OR perception)

AND TX (green space* OR park* OR green* OR forest* OR garden*)

AND TX (citizen* OR urban OR city OR cities OR metropolitan)

**LIVIVO (n=733 results)**

(sound* OR auditory OR birdsong* OR acoustic)

AND (mental OR "quality of life" OR well* OR restorat* OR stress OR relaxation OR satisfaction OR emotion* OR mood OR perceiv* OR recovery OR psychological OR comfort OR perception)

AND (green space* OR park* OR green* OR forest* OR garden*)

AND (citizen* OR urban OR city OR cities OR metropolitan)

**Medline (n=131 results)**

((sound*.tw. OR auditory.tw. OR birdsong*.tw. OR acoustic.tw.)

AND (mental.tw. OR "quality of life".tw. OR well*.tw. OR restorat*.tw. OR stress.tw. OR relaxation.tw. OR satisfaction.tw. OR emotion*.tw. OR mood.tw. OR perceiv*.tw. OR recovery.tw. OR psychological.tw. OR comfort.tw. OR perception.tw.)

AND (green space*.tw. OR park*.tw. OR green*.tw. OR forest*.tw. OR garden*.tw.) AND (citizen*.tw. OR urban.tw. OR city.tw. OR cities.tw. OR metropolitan.tw.))

**Embase (n=145 results)**

((sound*.tw. OR auditory.tw. OR birdsong*.tw. OR acoustic.tw.)

AND (mental.tw. OR "quality of life".tw. OR well*.tw. OR restorat*.tw. OR stress.tw. OR relaxation.tw. OR satisfaction.tw. OR emotion*.tw. OR mood.tw. OR perceiv*.tw. OR recovery.tw. OR psychological.tw. OR comfort.tw. OR perception.tw.)

AND (green space*.tw. OR park*.tw. OR green*.tw. OR forest*.tw. OR garden*.tw.)

AND (citizen*.tw. OR urban.tw. OR city.tw. OR cities.tw. OR metropolitan.tw.))
