# Supplementary material for: Exploring the relationship between mental health and urban green space soundscapes: A scoping review
Source: PLoS One. 2026 Mar 3;21(3):e0344125. doi: 10.1371/journal.pone.0344125 (PMC12956135; doi:10.1371/journal.pone.0344125)
Supplement: S1 Table — (PDF) [file pone.0344125.s002.pdf]

First Author (Z-A)	Sample Size	Population (age, gender, education, income)	Measurement tools for mental health outcomes	Assessment of soundscape elements	Applied data analysis
Zhu Y.	30	Mean age: 23.83 ± 2.28 years;<br>Gender: 43% male, 57% female;<br>College students	Short-version Revised Restoration Scale (SRRS) and a custom perceived health benefits scale	Soundscape elements were assessed by identifying 11 frequently occurring sounds, categorized as natural, human, mechanical, and cultural sounds. Participants used a 7-point Likert scale (Sound source identification (ISO/TS 12913-2, 2018) to rate the prominence of these sounds at each location, and soundscape perceptions were evaluated through indicators such as pleasantness, harmony, variety, and psychoacoustic parameters like loudness, sharpness, and roughness.	Spearman's rho correlation, Exploratory Factor Analysis (EFA), Confirmatory Factor Analysis (CFA), and Structural Equation Modelling (SEM) were applied to explore relationships between soundscape perception, perceived restoration, and health benefits.

Zhou Y.	901	Mean age: n. a.; 53.13% of respondents between 18–29 years;<br>48.32% with university degree, 50.56% less than 4,000 Yen income	Questionnaire surveys assessing citizens' emotional responses using a modified PANAS scale with five latent variables and 19 observed variables	Natural sounds (e.g., bird calls, wind), living sounds (e.g., conversation, laughter), mechanical sounds (e.g., traffic, construction noise); sound pressure level (SPL) measurement	Structural Equation Modelling (SEM) was used to test hypotheses about the relationship between sound types and emotional responses. Multigroup analysis to explore seasonal differences in soundscape effects
Zhao W.	240	Age: 20-39 years (45%);<br>Gender: 46% male, 54% female;<br>Mixed educational levels	Perceived Restorativeness Soundscape Scale (PRSS), stress previous month (in questionnaire about sociodemographic background)	Different sound types were evaluated (sparrow, woodpecker, magpie, crow) at various heights (0.5m, 2m, 4m).	Paired sample tests, Spearman’s correlation, factor analysis, non-parametric tests

Zhang T.	250	Age: the majority fall within the 18–30 age range; Gender: 42% male, 58% female; Most respondents were in good health, and park visits were usually with friends (24%), lover (21%), or alone (21%)	Likert scale for mental restoration (based on ART framework); emotional responses (comfort, happiness, sense of belonging, attraction) and behavioral activities (relaxation, social interaction, exercise, walking, art, family activities) were measured using five-point Likert scales for intensity and frequency	Auditory sensation measured using a Likert scale (natural sounds, sweet background music, happy people sounds (singing or playing instruments), quiet space (background city), and no traffic noise	Structural equation modeling (SEM), mediation effect analysis (bootstrapping 5000 samples)
Zhang Y.	204	Mean age: 24 years; Gender: 52.27% male, 47.73% female	PSS-14 for stress perception, PANAS for mood assessment, heart rate and Root Mean Square of	Natural (e.g., birdsong, water flow), mechanical (e.g., traffic noise), and social sounds analyzed quantitatively using a questionnaire	Multiple linear regression modeling; correlation analysis (Kolmogorov–Smirnov test, Pearson correlation)

			Successive Differences as physiological stress markers		
Yin	20	Mean age: n.a.; young adults aged 20-30;<br>Equal gender distribution	Russell's Circumplex Model; Likert scale-based questionnaire based on Perceived Affective Quality (PAQ) and ISO 2018.	Measurement of traffic, mechanical, anthropological and natural sounds (ISO 2018) doing soundwalks using a questionnaire-based onsite-survey (Likert-scale: agree-disagree)	Linear regression, correlation analysis, QGIS mapping
Wu	904	Mean age: n.a.;<br>Gender: 61.4% male, 38.6 % female;<br>SES: n.a.	PANAS (Positive and Negative Affect Schedule), Perceived Restorativeness Scale (PRS)	Sound quality satisfaction (SQS), sound pressure level (SPL)	Descriptive analysis, t-tests, ANOVA, Path Analysis (subset of SEM)
Tian	20	Mean age: 22 years;<br>Gender: 50% male, 50% female;<br>University students with normal hearing	Likert scale questionnaire based on ISO 12913-2, Perceived Affective Quality (PAQ)	SPL measurement, classification of sound sources (natural, mechanical, human), GIS-based spatial visualization of soundscapes	Correlation analysis for SPL and soundscape perception, GIS visualization

Payne	151	Mean age: 36 years;<br>Gender: 52% female, 48% male;<br>Mixed urban residents	Open ended questions; slightly adjusted Perceived Restorativeness Scale (PRS) used to assess restorative outcomes. Soundscape quality ratings captured perceptions of tranquility, calmness, and quietness. Coding of given reasons for park visits were also included to categorize answers.	Soundscapes were evaluated regarding calmness, tranquility, and quietness. Key elements included natural sounds (birdsong, leaves), mechanical sounds (traffic), and human activities (conversations). Quantitative and qualitative responses were recorded.	One way ANOVA, descriptive analysis and correlation analysis were applied to identify trends.
Marafa	445	Gender ratio and age breakdown not explicitly provided;<br>Socio-economic background collected	Self-reported tranquility ratings (1–10 scale), anxiety reduction scores (pre- and post-visit measurements),	Tranquility was influenced by greenery and reduced anthropogenic noise (e.g., traffic, conversations). The presence of natural soundscapes through greenery	Statistical analyses included ANOVA for comparing tranquility scores across green space types, correlation analysis between tranquility

		(recorded, details not provided).	and open-ended questions on tranquility factors	was critical for higher tranquility ratings.	and anxiety reduction, and post hoc Tukey tests for significant differences
Li W.	1161	Mean age: n.a., nearly 50% of the respondents between 26–45 years; Gender: 49.9% male, 50.1% female; Urban residents with mixed occupational and educational background	Perceived Restorativeness Scale (PRS)	Two-dimensional soundscape descriptors of Pleasantness-Eventfulness (PL-EV, ISO/TS 12913-2, 2018); HS6298 model multifunctional noise analyzer, bird songs, the sound of the wind, flowing water, and insect chirping; human sounds, including conversations, children playing, footsteps; mechanical sounds, traffic sounds, construction sounds, broadcast sounds	Shapiro-Wilk and Kolmogorov-Smirnov tests (for normality), Spearman non-parametric method; Pearson correlation; multiple linear regression; exploratory factor analysis was conducted on the acoustic environment model, utilizing Principal Component Analysis (PCA).
Li S.	41	Mean age: n.a.; Gender: 51.22 % male, 48.78% female;	Restoration Outcome Scale (ROS), Perceived Sensory Dimension Scale (PSD)	Focus on auditory components, such as natural sounds (birds, insects)	Partial Least Squares-Structural Equation Model (PLS-SEM)

		Students			
Lee J.S.L.	428	Mean age: n.a.; more than 50% between 26–45 years; Gender: male respondents; SES: n.a.	Subjective well-being of the soundscape experience is examined in the third section of the questionnaire, using single-item satisfaction and happiness questions	Perception of natural, human, traffic and other sound sources (constructions, ventilations, leaf blower, music, radio) using a five-point Likert scale, soundwalks, method A of the ISO/TS 12913-2, 2018)	Spearman’s rho correlation, ANOVA, Principal Component Analysis (PCA)
Lan	77	Mean age: 23.4 years; SES: n.a.; College students	Skin-conductivity (tension can be measured), heart rate, heart rate variability; study evaluates how landscape perception affects mental health using indicators such as mental vigor, stress relief, emotional calmness, and attention restoration. The questionnaire consists of	Sound environment monitoring, artificial sounds (human activity, mechanical sound) and natural sounds (geophysical sound, biological sound)	Stepwise regression analysis

			three parts: demographic information, perception of 15 typical forest sounds measured by frequency and intensity scores, and psychological evaluation using a seven-point Likert scale.		
Korpilo	45	Mean age: n.a., 24.4 % of respondents between 25–34 years;<br>Gender: 26.7 % male, 71.1 % female;<br>SES: 57.8 % with master's degree	Electrodermal activity (EDA) using smart rings; Restoration Outcome Scale (ROS), questionnaires with focus on mental health, pleasantness of audio-visual environment	Sound Source identifications using soundwalks (ISO/TS 12913–2, 2018), Normalized Difference Soundscape Index (NDSI); participants' subjective evaluation of pleasantness and appropriateness, Perceived Soundscape Quality (PSQ)	Generalized Linear Mixed Models (GLMM), Kruskal-Wallis tests, and linear regression
Jin	30	Mean age: n.a; range: 20–30 years;	Pleasure, calmness and satisfaction as emotional	Soundwalks and questionnaire (five-point Likert-scale about perceived	Shapiro-Wilk and Kolmogorov-Smirnov tests

		Gender: 50% Male, 50% Female;<br>SES: Mixed, recruited from local communities	evaluation indicators; all collected through questionnaire	soundscape as well as sound source categorization)	(for normality, correlation analysis and multiple regression models to explore relationships between audiovisual factors and outcomes
Jaszczak	40	Mean age: n.a., (aged 20–55 years);<br>Gender: n.a.;<br>20 experts (landscape architects and planners, 16 women and 4 men);<br>SES not specified	Mental mapping and qualitative interviews	Sound pressure levels (SPL) measurements only	SPL comparisons using paired t-tests; (qualitative analysis of mental mapping and interview data)
Herranz-Pascual	53	Mean age: 42.3 years (range: 19–75);<br>Gender: 54% women, 46% men;	CITI-SENSE questionnaire assessing emotional states with four basic emotions (happiness, calm, anger,	Ad-hoc questionnaire using semantic differential scale for soundscape (pleasantness, calmness, naturalness, etc.) based on ISO/TS 12913:2;	ANOVA, Pearson correlations, multiple linear regression models to assess predictors of emotional

		SES: mix of secondary and university education, 40.4% employed	sadness), assessed in the beginning and in the end of questionnaire	objective sound measurements using smartphones and microphones, acoustic comfort using a five-point ordinal scaled item	responses, Brown-Forsythe robust test, Kolmogorov-Smirnov test
Guo Y.	266	Mean age: n.a., 46% of respondents between 18–30 years;<br>Gender: 46.24% male, 53.76% female;<br>SES: education levels ranged from junior high school to postgraduate	Perceived Restorativeness Soundscape Scale (PRSS), Self-Assessment Manikin (SAM) scale for emotional response (pleasure, arousal and control)	Three different sound source types were assessed: natural sound (leaves, water, bird song), artificial equipment sound (traffic, broadcast music, construction site) and human activity (talk, footsteps, floor sweeping)	Spearman's correlation, Kruskal–Wallis test; multiple linear regression analysis
Guo X.	419	Mean age: n.a., 46% of respondents between 25–40 years;<br>Gender: male (39.9%), female (60.1%);	Soundscape Pleasantness (SSP), Soundscape Eventfullness (SSE) and Perceived Restorativeness Soundscape Scale (PRSS)	Two dimensions were assessed: soundscape pleasantness (e.g. harmonious, comfortable) and soundscape eventfulness (e.g., vivid, various)	Structural Equation Modelling (SEM), confirmatory factor analysis, hypothesis path analysis, mediating effect

		SES: various educational backgrounds ranging from high school to master's degree			analysis, and multi-group analysis
Guo X.	419	Mean age: n.a., 46% of respondents between 25–40 years; Gender: male (39.9%), female (60.1%); SES: various educational backgrounds ranging from high school to master's degree	Perceived Restorativeness Soundscape Scale (PRSS)	Two soundscape dimensions were assessed:<br>- Pleasantness: natural sounds (birdsong, water flow) associated with calmness.<br>- Eventfulness: dynamic sounds (human activity, laughter) linked to energy and engagement	Structural Equation Modeling (SEM), mediation/moderation analysis to explore relationships between soundscape characteristics, visitor behavior, and SRE
Fisher	449	Mean age: n.a.; mixed age range (mostly under 45 years, 71.9% of participants between 18–45 years);	Self-reported surveys, including the Perceived Restorativeness Scale (PRS) and psychological wellbeing	Soundscape characteristics were assessed by field acoustic recordings and participant perceptions of biophonic (natural) and anthrophonic (human-made) sounds through	G-tests to confirm sample representativeness, Kruskal-Wallis tests for numerical comparisons, and chi-squared tests for categorical data.

		Gender: 55% male, 45% ; female; Sociodemographic background representative, except for income and education; mostly alone (70%), visiting daily (49%)	scales (PANAS; STAI) assessing affect and anxiety	Normalised Difference Soundscape Index (NDSI)	Linear mixed-effect models were applied to examine how perceived sound enjoyment, bird species richness, naturalness, and safety concerns influenced wellbeing outcomes like positive affect and anxiety. Mediation models were used to explore the role of perceived restorativeness, with site as a random effect and landcover type as a fixed effect. Bootstrapping (5000 simulations) was used to estimate mediation effects and ensure robustness
