# Supplementary material for: Exploring the relationship between mental health and urban green space soundscapes: A scoping review
Source: PLoS One. 2026 Mar 3;21(3):e0344125. doi: 10.1371/journal.pone.0344125 (PMC12956135; doi:10.1371/journal.pone.0344125)
Supplement: S2 Table — (PDF) [file pone.0344125.s003.pdf]

First Author (Z-A)	Key findings	Limitations	Practical recommendations
Zhu Y.	Natural sounds, such as birdsong, running water, and wind-blown leaves had the most positive effects on soundscape perception, restorative perception, and health benefits. Birdsong was strongly associated with pleasantness, promoting emotional recovery and cognitive focus, while water sounds reduced noise annoyance and increased relaxation. The study highlights that restorative perceptions mediate the link between pleasant soundscapes and health benefits, suggesting that enhancing natural soundscapes in urban green spaces can improve mental well-being.	Focus on sound only; lacks multi-sensory inputs. Self-reported data may have biases. Limited sample (10 plots, college students). Conducted only in summer; not generalizable across seasons.	Include multi-sensory inputs and objective measures. Use VR for controlled experiments. Expand to diverse parks, populations, and seasons to improve forest therapy design.
Zhou Y.	Natural sounds boost positive emotions, especially in summer, but have little impact on reducing negative emotions; living sounds like laughter and children	Limited to parks in Beijing; more diverse green spaces could be studied; potential bias in self-reported emotions	Enhance natural soundscapes, manage mechanical sounds, separate living sound zones, introduce soundscape professionals

	playing generally promote relaxation and positive feelings.<br>Mechanical sounds: construction and traffic noises increase negative emotions and reduce well-being.<br>Seasonal differences: summer sounds are more calming, while winter winds can cause irritation. Greenery can improve sound perception and emotional responses.		
Zhao W.	Different types of bird song influence perceived restorativeness (PRSS), with woodpecker and sparrow sounds scoring highest for comfort and preference, while crow sounds score the lowest.<br>Seasonal differences affect soundscape perception, with summer soundscapes being more restorative due to higher sound comfort and preference, particularly for fascination;	Use of artificial sounds via stereo; limited generalizability due to controlled environment	Promote biodiversity in urban parks, design spaces to integrate natural soundscapes at optimal heights

	Birdsong height impacts evaluations, with the highest PRSS scores at four meters, where birdsong integrates naturally with the environment.<br>Demographic factors such as age, education, and stress levels affect PRSS. Older participants tend to give higher ratings when birdsong occurs at lower heights.		
Zhang T.	Multi-sensory perception (auditory, visual, and tactile) influences mental restoration by emotional responses and behavioral activities. Among the senses, auditory sensation had the most substantial impact on mental restoration.	The study focused only on main effects of the three sensations on mental restoration, without exploring interactions between the senses.	Emphasize auditory sensations in park design
Zhang J.	Natural sounds (e.g., birdsong and leave rustling) significantly improved HR and RMSSD, indicating stress recovery; mechanical sounds impeded recovery; social sounds had moderate effects.	Focus on a single park, leading to regional limitations in its conclusions. Group of participants was not homogenous. Ecological factors like temperature, biodiversity,	Urban Park design should focus on reducing traffic noise using vegetation barriers and increase natural sounds like water flow and birdsong to enhance health restoration. Soundscapes should be carefully managed by masking

		and seasonal variations were not considered.	unwanted sounds with soothing music or natural sound elements and improving park maintenance to create a calming acoustic environment.
Yin	Natural sounds and landscapes positively influenced emotional and behavioral responses. Grasslands and woodlands promote recreational activities, especially when combined with water features. Hardscapes and traffic sounds can reduce emotional satisfaction.	Limited sample size, conducted only in summer, may not represent broader population reactions, young adults' perceptions may not be representative to all age groups, strong summer heat may cause biased responses.	Urban green space design should incorporate diverse natural sounds and landscapes to enhance positive emotional experiences.
Wu	Visitors` emotions are more influenced by physical and psychological factors than personal characteristics. Thermal comfort has the largest impact on emotions, followed by sound and air quality. Restorativeness played a key role in improving emotional outcomes.	The study was conducted only in summer, and seasonal effects were not explored. The sample was limited to one city in China.	Urban green space designs should prioritize enhancing air quality, soundscapes, thermal comfort, and restorative environments by maintaining high levels of greenery, biodiversity, and natural elements. Practical measures

			include incorporating water bodies and colorful plants, planting trees with dense crowns for shade and dust reduction and using reflective materials for roads and plazas to mitigate heat.
Tian	Landscape elements influence soundscape perception, water bodies and vegetation reduced SPL differently; natural sounds (birdsong, water flow) improved relaxation and pleasure perception.	Limited generalizability due to small and homogenous sample; demographics restricted to university students.	Increase natural sound sources in parks, optimize vegetation placement to reduce sound pressure levels, enhance visual landscapes to improve soundscape perception.
Payne	Participants reported higher restorative outcomes in areas perceived as calm and tranquil. Soundscapes dominated by natural sounds provided the greatest benefits, while mechanical sounds (e.g., traffic) reduced perceived restoration. Perceived restoration in the park was higher than in the garden and the square.	Limited geographic scope to three cities in the UK; variability in quiet area characteristics not fully controlled	No recommendations explicitly stated.

Marafa	Country parks had the highest tranquility ratings and anxiety reduction. Urban open spaces scored the lowest regarding tranquility. Factors enhancing tranquility included fewer vehicles, presence of greenery, and reduced human disturbances. Tranquility was linked to reduced anxiety and improved emotional well-being.	Not reported. Limited generalizability due to the focus on Hong Kong.	Design of areas as restorative environments according to the human preferences of the visual and sonic environment by various stakeholders, e.g., development of tranquility trails and mapping of tranquility contours of a certain area.
Li W.	Bird songs and water sounds have a significant positive effect on perceived restoration, while Humans sounds and mechanical sounds had a significant negative effect on perceived restoration. Age is positively correlated with perceived restoration. Furthermore Pleasantness also had a positive effect on perceived restoration. Social sounds had mixed effects, with positive impacts in some contexts (e.g., children playing) and negative impacts in others (e.g., loud conversations). There is interaction with visual components, for example the presence of trees,	Interaction with other factors like smell. Observational study influenced by subjectivity of researchers; cultural/geographical specificity. No physiological health outcomes were measured.	Urban parks can be restorative by reducing noise and enhancing natural sounds like birdsong. The allocation of dedicated quiet zones and activity areas helps meet diverse needs without compromising tranquility. Thoughtful landscaping and acoustic planning improve relaxation and social engagement, promoting a balanced

	grass and water surfaces can enhance the pleasantness of soundscape experience. Effects of sound sources were masked by interaction with visual settings.		connection between nature and urban life.
Li S.	The urban forest environment significantly influences psychological restoration via environmental perception, with visual elements ranked first in importance and auditory elements second.	Research only conducted in Spring; small sample size	3S technology, virtual reality equipment, and eye trackers can be utilized to do virtual experiments for real-time monitoring and efficient data collecting about the urban forest environment and human behaviors, grounded in a thorough consideration and configuration of multi-sensory perception.
Lee J.S.L.	Soundscape characteristics (pleasantness and eventfulness) varied across parks; natural sounds positively influenced well-being, traffic sounds negatively influenced well-being. The results for human activities were mixed throughout the parks.	Objective measurements may not significantly predict subjective well-being effects from park visitations; cultural/geographical specificity	Enhance natural sounds in parks, reduce traffic noise, and design tranquil zones for optimal well-being outcomes, further studies should also focus on visual

			effects of landscapes together with soundscapes.
Lan	The study found that urban forests, especially waterfront forests, significantly reduce stress levels and improve mood, supporting the Stress Recovery Theory (SRT) and Attention Restoration Theory (ART). Waterfront forests, due to their natural water sounds and open vistas, offer profound psychological restoration benefits by masking urban noise and enhancing tranquility. However, the study highlights that anthropogenic disturbances, such as noise pollution and high visitor traffic, can reduce restorative effects, suggesting the need for buffer zones and noise-reduction strategies in urban forest designs to maximize mental health benefits.	The study's sample size is limited to three forests in Fuzhou, which may not be representative of other regions or urban forest types; research focuses on short-term physiological and psychological responses, with a need for future studies on long-term exposure; study relies on self-report questionnaires, which could introduce subjectivity, suggesting the need for more extensive survey methods in the future.	Future research could explore responses from different cultural, age, and gender groups to improve urban forest designs that meet diverse needs. Additional studies could investigate the impact of community engagement, educational programs, and recreational activities on the audiovisual environment and public health. These insights can help guide the creation of healthier and more sustainable urban environments.
Korpilo	Both physiological and psychological restoration were greater in forest and beach environments compared to the urban park. Restoration effects were linked to higher	Limited sample size, lack of control over some environmental variables, and potential unmeasured participant	Advocate for the integration of diverse natural elements in urban planning to enhance restorative potential; emphasize

	natural sound dominance (biophonic sounds). More anthropogenic and traffic noise in the urban park. Other visitors had positive and negative moderating effects.	factors like medication use. Physiological restoration is affected by participants' "stress baggage" i.e., level of stress before entering the place.	accessibility and proximity of green and blue spaces for urban populations (3-30-300 rule of thumb: 3 trees to see from home, 30% tree canopy cover in their community, not more than 300m away from green spaces).
Jin	Lower SPLs (<77 dB) were positively correlated with satisfaction and pleasure, natural sounds, such as birdsong and water flow, enhanced calmness and vitality. Pavement visibility and mechanical noise had negative impacts on emotional responses.	Underrepresentation of more enclosed and vegetated areas, requirement for 300m distance between points could result in exclusion of smaller parks, small sample size; results limited to a single urban park in Chengdu, self-reported data bias.	Reduce mechanical noise levels in urban parks, with 77 dB as a critical threshold; enhance the visibility of natural landscapes, such as woodlands and water bodies, while minimizing paved areas; Incorporate soundscape planning into urban park design to boost emotional recovery and vitality.
Jaszczak	Central areas of the park with dense vegetation were rated highest for tranquility. Seasonal variation	Small sample size, limited generalizability due to homogenous sample, self-selection bias:	The proposed modifications include the incorporation of diverse plant species, a green wall, and a water curtain, designed

	influenced soundscape perceptions, with leafy conditions reducing noise significantly.	recruitment via university student channels.	not only to mitigate noise but also to enhance the well-being, as well as the mental and physical health, of campus users.
Herranz-Pascual	Green urban spaces with pleasant and natural soundscapes reduced negative emotions and stress, while slightly increasing happiness and calm. Environmental and acoustic comfort strongly predicted emotional restoration.	No statistical significance for effect on positive emotions, small sample size; limited diversity of urban spaces studied; complexity of subjective data collection method, potential observer bias due to the presence of researchers, limited generalizability due to specific urban settings.	Incorporate soundscape considerations into urban design. Create quieter, greener urban areas with diverse natural sound sources (e.g., water, birdsong).
Guo J.	Older participants rated natural sounds higher for stress relief, participants with higher education perceived natural soundscapes less restorative; differences between stress relief in men and women; birdsong, flowing water,	Relied on subjective measurements; lacked physiological validation of stress relief; limited generalizability to broader urban contexts, possible	Enhance natural soundscapes (e.g., birdsong, running water); plan restorative spaces catering to diverse demographic groups; incorporate

	and breeze sounds provided the strongest restorative effects; More frequent park visits increased soundscape sensitivity (more nervous and excited about man-made and artificial sounds, calmer about natural sounds).	sampling bias from participant self-selection; findings are location-specific.	soundscape design into urban forest park planning.
Guo X.	Soundscape pleasantness had the most significant positive effect on perceived restoration. Visual landscape characteristics had a mediating role, enhancing the perceived restoration through audio-visual interactions. Age was the most influential demographic factor (people below 40 profited from pleasant soundscapes, while people over 40 years old preferred eventful soundscapes), followed by gender. Occupational and educational background less important.	Lack of exploration of the physical environment's direct influence on perceptions, limited visitor characteristics considered, and the need for more detailed age segmentation.	Design parks with harmonious soundscapes and visually appealing landscapes to enhance perceived restoration. Consider visitor demographics such as age and gender in park planning. Integrate natural elements to improve audio-visual coherence for higher restorative benefits.
Guo X.	Pleasant soundscapes were strongly linked to static behaviors (e.g., sitting, reflecting) and greater restoration; Eventful soundscapes encouraged dynamic behaviors (e.g., jogging, socializing) but had a weaker restorative	Small geographic scope limited to five parks in Fuzhou; cross-sectional design limits causal interpretation;	Static Behavior Areas: Prioritize soothing natural sounds (e.g., birdsong, water) to encourage longer stays and enhance restorative effects; Dynamic

	effect; Visitor behavior acted as a mediator between soundscape and SRE.	reliance on self-reported data introduces potential biases.	Activity Areas: Incorporate energetic and diverse sounds to create a stimulating and engaging atmosphere; Movement/Transition Areas: Ensure seamless sound transitions with fluid auditory stimuli to avoid abrupt changes as visitors move between spaces.
Fisher	Spaces with higher perceived biodiversity, natural sounds (e.g., birdsong), and safety were linked to greater psychological restoration and reduced anxiety; Biophonic sounds (e.g., birdsong) had a more significant positive impact on perceived restorativeness than anthrophonic sounds (e.g., human noise). This results in improved well-being.	Limited generalizability due to single specific city, self-reported data, selection bias toward participants more interested in nature.	Further multidisciplinary research is required for where objective measures get further developed.
