# Supplementary material for: Exploring the relationship between mental health and urban green space soundscapes: A scoping review
Source: PLoS One. 2026 Mar 3;21(3):e0344125. doi: 10.1371/journal.pone.0344125 (PMC12956135; doi:10.1371/journal.pone.0344125)
Supplement: S1 File — (PDF) [file pone.0344125.s004.pdf]

Exploring mental health effects of urban green space soundscapes: a scoping review protocol

Ahmadi E[1,2], Baierl S[1,2], Coenen M[1,2]

[1] Institute for Medical Information Processing, Biometry, and Epidemiology – IBE, Chair of Public Health and Health Services Research, LMU Munich, Munich, Germany

[2] Pettenkofer School of Public Health, Munich, Germany

Introduction

In the past and present, the detrimental health effects of noise were investigated and discussed thoroughly in a variety of contexts and settings. While there is a broad range of scientific literature on noise pollution and its various adverse health effects, the scope of the research is predominantly limited on noise as a one-dimensional variable which focuses solely on negative health outcomes (Schulte-Fortkamp et al., 2023, pp. 4-5). This narrow perspective created a growing motivation and interest in investigating noise for its complex effects on humans. The resulting paradigm shift emerged in form of research which can be described by the umbrella term "Soundscape". Soundscape research is a multidisciplinary field, where the multi-layered acoustic perception of humans is in the focus. Soundscape research emphasizes human perception and interaction with acoustic environment, proposing that sound elements in urban green spaces may positively influence mental health by providing restorative experiences.

A prominent area of current soundscape research is the examination of these health outcomes in an urban context. The objective is oftentimes correlated with gathering crucial scientific insights for the creation of restorative green spaces in urban areas. This research focus is supported by the significant increase in the global urban population, from 0.75 billion in 1950 to 4.22 billion in 2018. It is estimated that by the mid-twenty-first century, 68% of the world's population will reside in urban areas (Kundu et al., pp. 13-49). Consequently, it is vital to develop future public health policy recommendations with the objective of mitigating the adverse health effects of urbanization on the urban population.

This scoping review will explore how specific soundscape components, such as natural sounds in urban green spaces, impact mental health outcomes. The mapping of evidence will inform public health strategies and urban planning, supporting mental well-being through the intentional design of restorative soundscapes in cities.

Objectives

The objective of this scoping review is to provide a comprehensive overview of the existing literature on soundscapes and their effects on human mental health embedded in an urban context. Specific aims include:

1. Classifying methodologies and study designs used in this domain.
2. Identifying which mental health outcomes are studied in connection with urban green space soundscapes.
3. Exploring the types of soundscape elements (e.g., birdsong, water sounds) and their correlation with mental health outcomes.

Methods

The proposed scoping review will be conducted in accordance with the updated JBI methodology for scoping review, furthermore the Preferred Reporting Items for Systematic Reviews and Meta-Analyses extension for Scoping Reviews (PRISMA-SCR) will be taken into account (Peters et al., 2020). The decision to conduct a scoping review is based on the objective of mapping the existing literature on a specific topic to identify key concepts and research conduct, rather than synthesizing the effects of a certain intervention (Munn et al., 2018).

Furthermore, the applied scoping review is based on a methodological framework proposed by Arksey and O'Malley (Arksey & O'Malley, 2005), considering later refinement suggestions by Levac et al. (Levac et al., 2010) and Daudt et al. (Daudt et al., 2013), as well as later on recommended enhancements by Westphaln et al. (Westphaln et al., 2021).

Initially, the framework will be constructed within five steps:

1. Identifying and specifying the research question
2. Identifying relevant literature
3. Selection of studies
4. Extraction, mapping and charting the data
5. Summarizing, synthesizing and reporting of the results

Step six included the integration of expert consultation, Arksey and O'Malley consider this step as an optional component (Arksey & O'Malley, 2005). This step is not going to be considered for this scoping review, due to the lack of resources and time.

1. Identifying and specifying the research questions

To conduct a scoping review, it should be first investigated what literature already exists on soundscapes and their effects on health. A PCC framework is used, to adhere to the JBI Manual suggestions and to obtain clarity in further steps of the scoping review process (Peters et al., 2020).

Category	Determinants
Population/Participants	Individuals residing in urban areas
Concept	Perception of soundscapes in urban green spaces as related to mental health
Context	Urban green spaces, including parks, greenways, and recreational areas

2. Identifying relevant literature

The literature search will be conducted through following databases: 1) Medline 2) Web of Science and 3) PsycInfo. The initial search strategy will be adapted through an iterative process pursued by the authors. Furthermore, literature will be also included manually through citation analysis. The time frame is set from 1985 to 2024, as the elusive nature of the soundscape-concept could have been investigated, without specifically referring to the term itself yet. The language of the papers is limited to English. The bibliography software in use will be EndNote (Version 21), duplicates will be removed manually and through EndNote. The proposed reconstruction of the PCC-Framework will be explained in the next step.

Table 1: Adjusted PCC-Framework

Category	Determinants
Population/Participants	
Concept	("Sound*" OR “soundscape*” OR "acoustic environment" OR “natural sound*” OR "auditory environment*" OR “birdsong”) AND ("Mental Health" OR "Quality of Life" OR "well-being" OR “well being” OR "stress" OR “relaxation” OR “restorative” OR “acoustic comfort”)
Context 1	("Parks, Recreational" OR "Green Space*" OR "green infrastructure*" OR park*)
Context 2	(“citizen*” OR “city” OR “cities” OR “metropolitan” OR “urban”)

3. Study selection

For the third step the PCC Framework will be adjusted – the context will be divided into two contexts. This decision was made due to an urban environment functioning more as a contextual frame than describing a distinct population.

For the screening process the review tool "Rayyan" will be used. The screening process will be carried out in two phases: for the first phase titles and abstracts will be screened. In the second phase, a full text screening with all included studies from the first phase will be conducted. All team members will test the explicitness of the inclusion and exclusion criteria individually in a blinded process in 50 selected references. The eligibility criteria will be adjusted, after discussing conflicts in the testing phase.

Table 2:

**Category**	**Inclusion Criteria**	**Exclusion Criteria**
Population	Studies focusing on adults of any age above 18 years	• Studies including children and adolescents<br>• Studies focusing on clinical populations and patients<br>• Studies including tourists of a city only
Concept	Studies investigating the perception of soundscapes related to mental health outcomes (well-being, stress reduction, relaxation, restoration); Research methods applied to assess perceptions, such as soundwalks, psychoacoustic measurements, surveys and interviews	• Studies measuring only physical noise levels through decibel levels<br>• Studies focusing on noise annoyance without emphasizing subjective perceptions of soundscapes<br>• Studies that do not measure any mental health outcomes related to soundscapes
Context 1	Studies conducted in green spaces including parks, forests, nature reserves and public gardens beside others<br><br>Studies conducted in at least one green space and grey spaces reporting the results of the green space(s) under consideration separately from the results of the grey space(s)	• Studies conducted in indoor environments (offices; hospitals, etc.)<br>•<br>• Studies conducted in architectural or built environments without green or natural space components (shopping malls, stadiums, etc.)<br>• Studies conducted in green spaces that are restricted outdoor areas (not accessible for the public);<br>• Studies conducted in grey spaces only

		• Studies conducted in grey and green spaces which do not report results of green spaces only
Context 2	Studies conducted in urban environments and metropolitan areas	• Studies conducted in rural areas<br>• Studies conducted in wilderness settings<br>• Studies conducted in non-urban green spaces (e.g., national parks, forests)<br>• Studies conducted in industrial areas

Study design

We will include research papers using any empirical qualitative and / or quantitative study design.

Type of publication

Publication of original research papers will be included.

Editorials, commentaries, letters to the editor, theoretical/discussion papers, methodological papers, study protocols and any kind of reviews will be excluded. Systematic and scoping reviews will be flagged during screening to check the reference lists for potentially primary studies of interest (snow balling).

Time and language of publication

There will be no restriction regarding the publication year of studies to be included. Only studies in English and German language will be included.

4. Extraction, mapping and charting the data

An Excel Spreadsheet will be used to extract data from the included studies. The data extraction framework includes general and specific information about the study related to the stated objectives and research questions (Arksey & O'Malley, 2005). It may be further refined iteratively by all team members during the review stage, updating the table accordingly through added parameters (Aromataris E. et al., 2024).

The general information section will contain the author(s), journal, year, country of origin, city (where the study has been conducted), objective, population (age, gender, socio-economic status (SES)), sample size and the methodology used, setting (see table 3).

The specific information section will include the following aspects: Type of urban green space, description of green (and grey) spaces, primary and secondary mental health outcomes, measurement tools for mental health outcomes, assessment of soundscape elements, use of soundscape standards, , data analyses applied, key findings, limitations, suggestions for future research (see table 4).

5. Summarizing, synthesizing and reporting of results

The extracted data will be presented in two tables, with the first table focusing on delivering a general overview of the main characteristics of the included studies (table 3). Table 4 is aiming to provide a more specified overview of the extracted literature in relation to objectives of the scoping review.

Table 3: General tabular overview of literature

First author	Journal	Year	Country of Origin	City	Objectives	Population	Sample size	Methodology	Setting	Key findings
…										
…										

Table 4 (divided into two tables due to number of rows): specified tabular overview of literature

First author	Age	SES	Type of urban green space	Primary mental health outcomes	Measurement tools for mental health	Studied soundscape element	Use of Soundscape Standards
…							
…							

Soundwalks and participatory approaches	Analytical approach	Key findings and conclusions	Limitations	Suggestions for future research	

Literature cited:

Aromataris E, Lockwood C, Porritt K, Pilla B, Jordan Z, editors. JBI Manual for Evidence Synthesis. JBI; 2024. Available from: https://synthesismanual.jbi.global.

Arksey, H., & O'Malley, L. (2005). Scoping Studies: Towards a Methodological Framework. *International Journal of Social Research Methodology: Theory & Practice*, *8*(1), 19-32. https://doi.org/10.1080/1364557032000119616

Daudt, H. M. L., van Mossel, C., & Scott, S. J. (2013). Enhancing the scoping study methodology: a large, inter-professional team's experience with Arksey and O'Malley's framework. *BMC Medical Research Methodology*, *13*(1), 48. https://doi.org/10.1186/1471-2288-13-48

Kundu, D., Sietchiping, R., & Kinyanjui, M. *Developing National Urban Policies Ways Forward to Green and Smart Cities* (1st ed.)

Levac, D., Colquhoun, H., & O'Brien, K. K. (2010). Scoping studies: advancing the methodology. *Implementation Science*, *5*(1), 69. https://doi.org/10.1186/1748-5908-5-69

Munn, Z., Peters, M. D. J., Stern, C., Tufanaru, C., McArthur, A., & Aromataris, E. (2018). Systematic review or scoping review? Guidance for authors when choosing between a systematic or scoping review approach. *BMC Medical Research Methodology*, *18*(1), 143. https://doi.org/10.1186/s12874-018-0611-x

Peters, M. D. J., Marnie, C., Tricco, A. C., Pollock, D., Munn, Z., Alexander, L., McInerney, P., Godfrey, C. M., & Khalil, H. (2020). Updated methodological guidance for the conduct of scoping reviews. *JBI Evidence Synthesis*, *18*(10), 2119-2126. https://doi.org/10.11124/jbies-20-00167

Schulte-Fortkamp, B., Fiebig, A., Sisneros, J. A., Popper, A. N., & Fay, R. R. (2023). *Soundscapes Humans and Their Acoustic Environment* (1st ed.). Springer International Publishing AG. https://ebookcentral.proquest.com/lib/kxp/detail.action?docID=7240923

Westphaln, K. K., Regoeczi, W., Masotya, M., Vazquez-Westphaln, B., Lounsbury, K., McDavid, L., Lee, H., Johnson, J., & Ronis, S. D. (2021). From Arksey and O'Malley and Beyond: Customizations to enhance a team-based, mixed approach to scoping review methodology. *MethodsX*, *8*, 101375. https://doi.org/https://doi.org/10.1016/j.mex.2021.101375
